# Supplementary material for: Immediate early gene kakusei potentially plays a role in the daily foraging of honey bees
Source: PLoS One. 2020 May 6;15(5):e0222256. doi: 10.1371/journal.pone.0222256 (PMC7202604; doi:10.1371/journal.pone.0222256)

**Fig S1: Gene expression profile for c-Jun (A), Dop1 (B), GluR (C), Erk7 (D) and 5-HT2α during the daily foraging of honey bees**


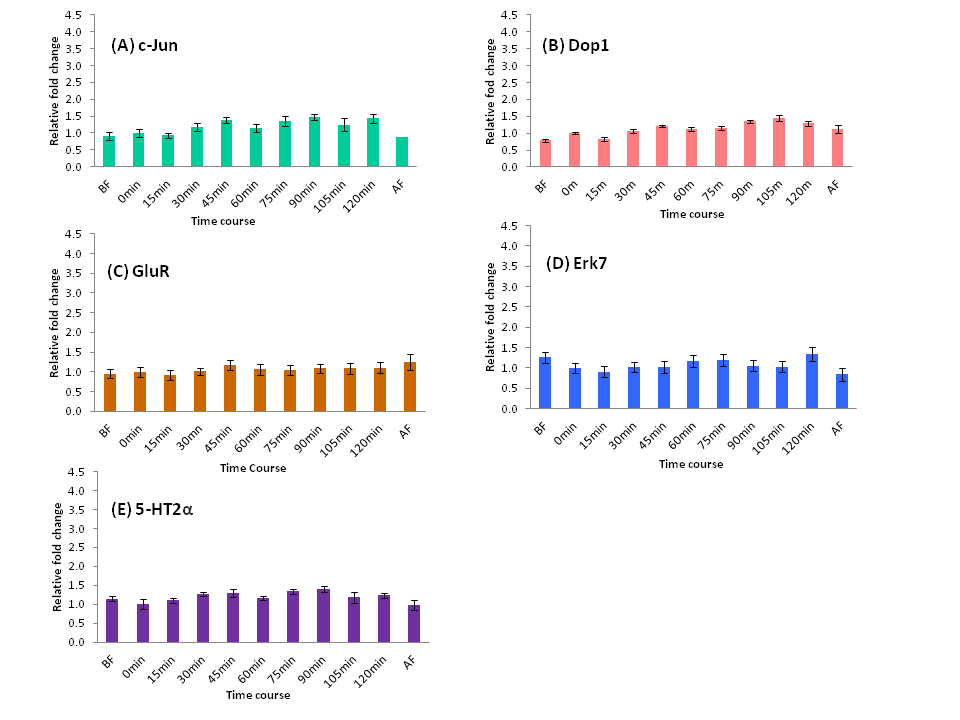

Supplement: S1 Fig — Gene expression profile for c-Jun (A), Dop1 (B), GluR (C), Erk7 (D) and 5-HT2α during the daily foraging of honey bees. (DOCX) [file pone.0222256.s001.docx]
