# Supplementary material for: Immediate early gene kakusei potentially plays a role in the daily foraging of honey bees
Source: PLoS One. 2020 May 6;15(5):e0222256. doi: 10.1371/journal.pone.0222256 (PMC7202604; doi:10.1371/journal.pone.0222256)
Supplement: S1 Table — (DOCX) [file pone.0222256.s002.docx]

**S1 Table. Summarized result for three replicate experiments of *kakusei***

| Time  points | *Kakusei* | | |
| --- | --- | --- | --- |
|  | Exp-1 | Exp-2 | Exp-3 |
|  |  |  |  |
| 0 vs 15 | < 0.0001 | < 0.0001 | < 0.0001 |
| 0 vs 30 | < 0.0001 | < 0.0001 | < 0.0001 |
| 0 vs 45 | < 0.0001 | < 0.0001 | < 0.0001 |
| 0 vs 60 | < 0.0001 | < 0.0001 | < 0.0001 |
| 0 vs 75 | < 0.0001 | < 0.0001 | < 0.0001 |
| 0 vs 90 | < 0.0001 | < 0.0001 | < 0.0001 |
| 0 vs 105 | < 0.0001 | < 0.0001 | < 0.0001 |
| 0 vs 120 | 0.0142 | < 0.0001 | 0.0023 |
| 15 vs 30 | < 0.0001 | 0.0034 | 0.0315 |
| 15 vs 45 | < 0.0001 | < 0.0001 | 0.0061 |
| 15 vs 60 | < 0.0001 | 0.0010 | 0.0033 |
| 15 vs 75 | 0.0006 | NS | NS |
| 15 vs 90 | NS | NS | NS |
| 15 vs 105 | NS | NS | NS |
| 15 vs 120 | NS | < 0.0001 | NS |
| 30 vs 45 | NS | NS | NS |
| 30 vs 60 | NS | NS | NS |
| 30 vs 75 | 0.0049 | NS | NS |
| 30 vs 90 | < 0.0001 | NS | NS |
| 30 vs 105 | < 0.0001 | < 0.0001 | NS |
| 30 vs 120 | < 0.0001 | < 0.0001 | 0.0001 |
| 45 vs 60 | NS | NS | NS |
| 45 vs 75 | 0.0039 | 0.0023 | NS |
| 45 vs 90 | < 0.0001 | 0.0002 | NS |
| 45 vs 105 | < 0.0001 | < 0.0001 | NS |
| 45 vs 120 | < 0.0001 | < 0.0001 | < 0.0001 |
| 60 vs 75 | NS | NS | NS |
| 60 vs 90 | 0.0241 | NS | NS |
| 60 vs 105 | < 0.0001 | < 0.0001 | NS |
| 60 vs 120 | < 0.0001 | < 0.0001 | < 0.0001 |
| 75 vs 90 | NS | NS | NS |
| 75 vs 105 | 0.0046 | < 0.0001 | NS |
| 75 vs 120 | < 0.0001 | < 0.0001 | 0.0006 |
| 90 vs 105 | NS | 0.0006 | NS |
| 90 vs 120 | 0.0002 | < 0.0001 | 0.0214 |
| 105 vs 120 | NS | NS | 0.0469 |
| Overall | < 0.0001 | < 0.0001 | < 0.0001 |

Statistics were performed using one way ANOVA with Turkey- Kramer multiple comparison test; BF: Before foraging; AF: Only the p values less than 0.05 are considered significant. NS stands for not significant (p >0.05).
