# Supplementary material for: Immediate early gene kakusei potentially plays a role in the daily foraging of honey bees
Source: PLoS One. 2020 May 6;15(5):e0222256. doi: 10.1371/journal.pone.0222256 (PMC7202604; doi:10.1371/journal.pone.0222256)
Supplement: S2 Table — (DOCX) [file pone.0222256.s003.docx]

**S2 Table. Summarized result for time trained feeding effect and unrewarded foraging on *kakusei* expression**

| Time points | Unrewarded | Rewarded |
| --- | --- | --- |
|  | p-value | p-value |
| 0 vs 15 | 0.0011 | < 0.0001 |
| 0 vs 30 | 0.0017 | < 0.0001 |
| 0 vs 45 | NS | < 0.0001 |
| 0 vs 60 | 0.0433 | < 0.0001 |
| 15 vs 30 | NS | 0.0195 |
| 15 vs 45 | NS | 0.0099 |
| 15 vs 60 | NS | NS |
| 30 vs 45 | NS | NS |
| 30 vs 60 | NS | NS |
| 45 vs 60 | NS | NS |
| Overall | 0.0011 | < 0.0001 |

Statistics were performed using one way ANOVA with Turkey- Kramer multiple comparison test; BF: Before foraging; AF: Only the p values less than 0.05 are considered significant. NS stands for not significant (p>0.05).
