# Supplementary material for: Immediate early gene kakusei potentially plays a role in the daily foraging of honey bees
Source: PLoS One. 2020 May 6;15(5):e0222256. doi: 10.1371/journal.pone.0222256 (PMC7202604; doi:10.1371/journal.pone.0222256)
Supplement: Attachment — Submitted filename: Response to the reviewers.docx [file pone.0222256.s004.docx]

*We are very grateful to the reviewers for the great effort in thorough reading of the manuscript and for providing valuable comments and suggestions that help to increase the quality of the manuscript.*

*The answers to the comments of the reviewers are given in the following:*

Reviewer #1: This study analyzed the expression time-course of immediate early gene (IEG) *kakusei*, which encodes a non-coding RNA, during repetitive foraging flights of honeybee workers. The analysis of neural IEGs including *kakusei* would give insight into molecular mechanisms underlying honeybee foraging. However, I think that the present study contains some serious problems. As the authors mentioned, the experimental design in this study is totally the same as it described in their previous study, which analyzed the other IEGs, egr-1 and Hr38. Although the authors assert that “the expansion of previous study”, I think that this is only “reuse”.

*Ans: The reviewer has pointed out very correctly. It is true that our experimental design in this study is same with that of the previous study. The samples that we used are also same; so, to say “reuse” is a correct word at this point. On the other hand, investigating kakusei and the other 5 new genes which we have not studied before to the same experimental design set up is what we mean to “expansion to the previous study”. To enclose reviewer’s comment we have inserted “the same samples have been reused in this study, in the method section line no. 107.*

*Thus we have also mentioned in discussion section a possible connection with kakusei and the two genes egr-1 and hr38 of the previous report.*

Furthermore, in conlusion section, the authors note that “The present finding on recently discovered IEG *kakusei* is an addition into the circle, thus increases the number of IEGs and ultimately more IEG choices available that could be used as search tool in finding molecular and cellular signaling underlying social behaviors, using honey bee foraging as model system.” However, some previous studies (e.g., Kiya et al. 2011, Ugajin et al. 2018, and Sommerlandt et al. 2018) have already discussed this point. It is difficult for me to find what this study newly provides to the field of behavioral and/or molecular biology of honeybees.

*Ans: We have adjusted the tone adding the first line in discussion section citing 4 references, and also modification is made in the conclusion section. Moreover we have cited more reference at the last part of the discussion section.*

I am also afraid that this manuscript contains a lot of overstating.

For example,

• Title: *kakusei* plays a role in ~

*Ans: We have added potentially to neutralize the overstatement, in the title.*

• Abstract: this study describes a fundamental role of the IEG *kakusei* ~

*Ans: We have substituted the word “fundamental” to “possible”, line no. 17.*

• Discussion: unraveling its role~, further examined the function of ~ This study does not perform any functional analysis of *kakusei*, but only examines the correlation between *kakusei* expression and time-course of foraging flight or food reward. The authors should entirely adjust the tone of their argument.

*Ans: We have also adjusted this tone by replacing “unraveling” with “suggesting” line no. 260, and the “function” by “possible role” line no. 267.*

Moreover, in discussion section, the authors mention as if *kakusei* involves in the process of associative learning (“this finding of *kakusei* involvement in learning and memory”). Shorter span upregulation of *kakusei* during the unrewarded foraging experiment only indicates the relationship between sustained *kakusei* expression and food reward. There are other possible factors resulting in the low level induction of *kakusei* during the unrewarded foraging, such as a lack of dance behavior, and lower motivation to fly toward the empty feeder. Further experiment (e.g. PER experiment) is needed to examine an involvement of IEG expression in associative learning.

*Ans: We have removed “associative” word from the entire text of the manuscript. We are very interested to do PER experiment. However, as all the authors currently work at different places on different projects, we are not able to predict when the facilities will be available to do this experiment. But this experiment is one of our priorities in the future plan.*

Minor points:

The results of statistical analysis described in TableS1 are not correctly reflected in Figure1.

*Ans: We have corrected in the figure.*

Although text says that there is no significant difference among three independent trials of before/after/start foraging experiment, Figure2B still have asterisk symbols.

*Ans: We have corrected. The result of the Fig 1 analysis was also mistakenly re-written there. What we wanted to write was the similarity between Fig 1 and Fig 2. We have reconstructed the sentence for more clarity.*

Gene name should be italicized (Title!).

*Ans: We have corrected.*

Von Frisch ---> von Frisch

*Ans: We have corrected.*

What does “09.00 hrs later” mean? 9 hours later? Or 9:00PM? Sampling time and procedure is very important for this study.

*Ans: We have changed to 18.00 hours, line no. 124.*

Reviewer #2: Review for: Immediate early gene *kakusei* plays a role in the daily foraging and learning of honey bees

This paper is about a non-coding gene, *kakusei*, that is found in the honey bee brain that seems to play a role in foraging and receiving reward. Generally, the authors collect bees after they have foraged for the day, before they have started foraging for the day, at different time points during their foraging day, at different time points during a time-shifted foraging day, and as they forage but get no reward. The authors find that this gene is upregulated as bees locate food, and if food continues to be found, but is quickly downregulated when there is no food, and is downregulated slowly if food is provided over time. It does not seem to be associated with time-shifted foraging. Overall, I believe the authors present very interesting results, and illustrate a relatively new area of behavioral genetics that deserves attention. However, the manuscript is confusing at times. Further, the authors overstate their results, as they do not test whether *kakusei* is related to associative learning, just with food reward during foraging. I have made suggestions for changes can be implemented to help the reader (especially readers who are not experts in this field) follow along.

*Ans: We appreciate for the kind words. We have removed “associative” word throughout the text of the manuscript.*

Introduction

Line numbers would be helpful for review.

*Ans: We have added line numbers this time.*

The introduction beginning with such a strong emphasis on waggle dance lead me to think it was going to be about waggling – although the authors do not examine the waggle dance in relation to *kakusei* regulation. The authors can give an overview of foraging behavior with short background of the waggle dance, but not such a large portion of the first paragraph.

*Ans: We have reduced the length of the sentence to about half from the previous one, line no. 35-36.*

I suggest breaking up the third paragraph. It has a lot of important information – especially for non-molecular biologists but is long. A natural break could be right before “In our recent work…”

*Ans: We have broken the paragraph right before “in our recent work” line no. line 65.*

From the MS: “The IEG c- jun (also known as jun-related antigen, jra in fruit fly) and egr-1 have been used as neuronal markers for the identification in honey bees of specific brain regions involved in the time memory as well as in, innate and learned behaviours [17,18,19].”

This sentence seems a little grammatically off to me – I think the comma before innate is unnecessary.

*Ans: We have removed the coma accordingly, line no. 75.*

Methods Methods are clear and concise.

*Ans: We appreciate for the complement.*

What brand and type of paint markers was used to mark bees?

*Ans: It is Uni POSCA Paint Markers (Uni Mitsubishi Pencil, UK). We have mentioned this also in the method section, line no. 118.*

How many colonies were involved in the experiment? Are they represented across all experiments and samples?

*Ans: We have use two colonies. The colony 1 was used for experiment 1 and 2, and colony 2 was used for experiment 3. For further clarity in the manuscript, we have also inserted this in the result section, line no. 177-179.*

Statistical analysis are effective for these data.

*Ans: We appreciate for the complement.*

Controls with other IEGs and orthologs are effective.

*Ans: We have done for c-jun another IEG but we found no difference. It is shown in supplementary figure. In our future work we will take it seriously to add more ortholog genes.*

Single-cohort age-matched bees would be best to use in experiments like these.

*Ans: We very much agree to it. It may be done by marking the bees from the larval stage. In our future experiment we will prioritize to it.*

Results

Please report statistical analysis performed with stated p-value.

*Ans: More statistics P-values are reported in the text, line no. 193, 228, 231, 243.*

From the MS: “This result demonstrates that Kakuei is involved in associative learning.”

You have not shown that this gene is upregulated in associative learning – another test of associative learning, such as PER, needs to be performed before that conclusion can be made. *Kakusei* seems to be involved with reward during foraging, but not necessarily with learning. Also *Kakusei* is spelled incorrectly in that sentence.

*Ans: We have removed “associative” word from the entire text of the manuscript. We are very interested to do PER experiment. However, as all the authors currently work at different places on different projects, therefore we are not able to predict when the facilities will be available to do this experiment. But this experiment is one of our priorities in the future plan. kakusei spelling is also corrected.*

Discussion

The link to associative learning has not been established with this study, and I suggest reducing the confidence of this language.

*Ans: We have removed “associative” word from the entire text of the manuscript.*

I agree that you have shown this, quoted from the MS: “This indicates that food reward is essential for the increase and sustaining of higher *kakusei* levels during foraging,…”

I do not agree that you have shown this: “…thus underlying the role of *kakusei* in associative learning during foraging.” You have not shown that honey bees are in fact learning during your assay.

*Ans: We have removed “associative” word from the entire text of the manuscript.*

*The feeder plate usually contain feeder, but bees still arrived and landed on the feeder plate even when the empty plate was placed. We paint marked the bees at the first arrival, and they still continue to come to the feeder though fewer and fewer, they might be expecting they would be rewarded, thus we were able to collect the samples until 60min, without rewarding food. Since, we observed significantly increased kakusei level from t0 to t15, but reduced after 15min as there was continuous unrewarded of food, we assumed that the bees were motivated to food reward which they experienced previously. If this explanation is still unsatisfactory, we will consider removing learning also from the entire manuscript.*

One experiment I would love to see to disentangle whether *kakusei* is upregulated when bees receive a reward or have learned is by doing PER on age matched foragers in the lab that haven’t foraged – and maybe to even see if it’s associated with strength of reward. It is a very easy test and would allow you to make the conclusions about learning.

*Ans: We have removed “associative” word from the entire manuscript. We are very interested to do PER experiment. However, as all the authors currently work at different places on different projects, therefore we are not able to predict when the facilities will be available to do this experiment. But this experiment is one of our priorities in the future plan. kakusei spelling is also corrected.*

From the MS: Thereby the hidden mechanisms of social behavior in honey bees could be opened up and mapped which could be further translated to higher animals and man [28].

Again, some of the language is unclear: Mechanisms are not hidden – aspects to gene function like non-coding regions are just now being discovered with relatively new techniques, which is really cool!

*Ans: We have removed “hidden”. We appreciate for the complement.*

Also, this hierarchical language of "higher animals" is not helpful in a scientific paper – “cognitively complex” would be a more accurate way to describe some animals (although bees are very cognitively complex). Also please use “humans” instead of “man”, although it is redundant after stating more cognitively complex animals.

*Ans: We have followed the good advice and changed accordingly, line no. 307.*

Figures

Figures are effective and relatively clear.

*Ans: We appreciate for the complement.*

In figure 2, I suggest using more descriptive X axis labels – I had to go back to the results to remind myself which experiment was which (although they are not clearly labeled experiment 1,2, and 3 there either). I suggest: During Foraging, Unrewarded Foraging, Time-Shifted Foraging. A and B labels are also missing.

*Ans: We have made it clearer in the legends to the figure and also we have changed the color pattern for easier viewing. We have also labeled experiment 1, 2 and 3 more clearly on the figure and the same is reflected on the results. We have also labeled A and B in Fig 2 which was missing earlier.*

For all figures, do the colors mean anything? I suggest to color-code by experiment, and keep it consistent though the two figures. Also explicitly state this in figure legends

*Ans: We have changed the color pattern in the Fig 1, three different colors for three experiments, same color for both A and B for each experiment. We have also mentioned color representations in the figure legends.*
