# Supplementary material for: Immediate early gene kakusei potentially plays a role in the daily foraging of honey bees
Source: PLoS One. 2020 May 6;15(5):e0222256. doi: 10.1371/journal.pone.0222256 (PMC7202604; doi:10.1371/journal.pone.0222256)
Supplement: Attachment — Submitted filename: Response to the Reviewers 3.docx [file pone.0222256.s005.docx]

*Dear Editor,*

*We are very grateful to you and to the reviewers for providing valuable comments and suggestions further that help to increase the quality of the manuscript.*

*The answers to the comments of the reviewers are given in the following:*

Reviewer #1: The authors generally addressed my comments except for PER experiment. But they carefully adjusted the tone related to the role of kakusei for learning and memory throughout the revised manuscript. Unfortunately, there are still inconsistency between Figure1 and TableS1. For example, regarding experiment 1A, are there significant differences in kakusei expression only “t0 vs t15” and “t15 vs t30”? Please correct Figure1 precisely.

*Ans: PER has been addressed in line no. 284-285 in this revision. Regarding the placing of asterisk symbols for all the significant differences in the figure 1, we have considered only the adjacent time points such as t0 vs t15” and “t15 vs t30, as noted by the reviewer; that is, we have not shown all the significant differences on the figure. This is for the reason that we wanted to show a clean figure as per the need of the description in the result and for further detail presentation of all the significant differences, Table S1 has been created. Nonetheless, we have also tried following the suggestion of the reviewer. However, we have kept the figure without changing the earlier form, because of the congestion of the lines, as shown below*

Reviewer #2: The explanation of learning is still not satisfactory. The authors have not fully tested the hypothesis that expression of kakusei is associated with learning. Changes in expression seen in this article could be due to a number of things – such as sensory reception of food or floral cues, digestion, navigation, etc. – which is still interesting! BUT much more work has to be done to link kakusei expression to learning. It is fine to propose in the intro and discussion, but it’s not explicitly tested here. I highly recommend removing any language about learning until it is explicitly tested. The figures also seem to be low resolution – some of the letters and shapes are difficult to read.

*Ans: We have diluted about the learning in the discussion to a great extend and we have added our future work plans to give a conclusive comment on it. The changes are made at line nos., 272-274, 282-286 and 300-302. We are also very grateful to the editor’s advice in this regard. We would like to do further changes if it is needed.*

*We have made the figures more clear this time.*
