# Supplementary material for: Immediate early gene kakusei potentially plays a role in the daily foraging of honey bees
Source: PLoS One. 2020 May 6;15(5):e0222256. doi: 10.1371/journal.pone.0222256 (PMC7202604; doi:10.1371/journal.pone.0222256)
Supplement: Attachment — Submitted filename: Response to the Reviewers 4.docx [file pone.0222256.s006.docx]

*Dear Editor,*

*We are very grateful to you and to the reviewers for providing valuable comments and suggestions and advices that help to increase the quality of the manuscript for the publication in your reputed journal PLOS ONE.*

*The answer to the comment of the reviewer is given as below:*

Reviewer #2: The title still contains the word "learning" which should be removed to reflect the changes in the manuscript.

Edotor: Please remove the words "and learning" from the manuscript title

*Ans: “and Learning” have been removed from the manuscript title.*
